# Testosterone and Androgen Receptor Sensitivity in Relation to Hyperactivity Symptoms in Boys with Autism Spectrum Disorders

**DOI:** 10.1371/journal.pone.0149657

**Published:** 2016-02-24

**Authors:** Anna Pivovarciova, Jaroslava Durdiakova, Katarina Babinska, Aneta Kubranska, Lenka Vokalova, Gabriel Minarik, Peter Celec, Marianna Murin, Daniela Ostatnikova

**Affiliations:** 1 Academic Research Center for Autism, Institute of Physiology, Faculty of Medicine, Comenius University, Bratislava, Slovakia; 2 Department of Molecular Biology, Faculty of Natural Sciences, Comenius University, Bratislava, Slovakia; 3 Institute of Molecular Biomedicine, Faculty of Medicine, Comenius University, Bratislava, Slovakia; 4 Department of Child and Adolescent Mental Health, Social Communication Disorders Clinic, Great Ormond Street Hospital for Children, London, United Kingdom; University of Jaén, SPAIN

## Abstract

**Introduction:**

Autism spectrum disorders (ASD) and hyperactivity symptoms exhibit an incidence that is male-biased. Thus androgen activity can be considered a plausible biological risk factor for these disorders. However, there is insufficient information about the association between increased androgen activity and hyperactivity symptoms in children with ASD.

**Methods:**

In the present study, the relationship between parameters of androgenicity (plasmatic testosterone levels and androgen receptor sensitivity) and hyperactivity in 60 boys (age 3–15) with ASD is investigated. Given well documented differences in parent and trained examiners ratings of symptom severity, we employed a standardized parent`s questionnaire (Nisonger Child Behavior Rating Form) as well as a direct examiner`s rating (Autism diagnostic observation schedule) for assessment of hyperactivity symptoms.

**Results:**

Although it was found there was no significant association between actual plasmatic testosterone levels and hyperactivity symptoms, the number of CAG triplets was significantly negatively correlated with hyperactivity symptoms (R^2^ = 0.118, p = 0.007) in the sample, indicating increased androgen receptor sensitivity in association with hyperactivity symptoms. Direct trained examiner´s assessment appeared to be a relevant method for evaluating of behavioral problems in the investigation of biological underpinnings of these problems in our study.

**Conclusions:**

A potential ASD subtype characterized by increased rates of hyperactivity symptoms might have distinct etiopathogenesis and require a specific behavioral and pharmacological approach. We propose an increase of androgen receptor sensitivity as a biomarker for a specific ASD subtype accompanied with hyperactivity symptoms. Findings are discussed in terms of their implications for practice and future research.

## Introduction

Autism spectrum disorders (ASD) are a set of heterogeneous neurodevelopmental conditions characterized by early-onset difficulties in social communication and unusually restricted, repetitive behavior and interests. The worldwide prevalence is about 1% [1]. A considerable percentage of children with autism spectrum disorder (ASD) also present attention deficits and hyperactivity symptoms often reaching the threshold for an ADHD clinical diagnosis (attention deficits/hyperactivity disorder) [2, 3]. Only recently, the American Psychological Association approved diagnosing co-morbid disorders such as ADHD along with ASD [4]. Both conditions can have a large negative impact on the daily life of affected individuals and their families, in particular when both conditions co-occur [5–7].

Recently released DSM 5´s criteria for ASD have been considered to be a useful framework to increase the homogeneity of research samples and encourage researchers to identify possible subtypes within ASD [4, 8, 9]. In a study conducted by Lecavalier [10] cluster analysis revealed several subtypes within ASD based on the behavioral/emotional problems, one of the subtypes characterized by increased rates of hyperactivity rated by teachers. ASD accompanied by hyperactivity might be one of the subtypes within the spectrum, characterized by distinct biological underpinnings and requiring specific behavioral and pharmacological approaches in comparison to the other subtypes within the spectrum. A better understanding of the etiology of ASD- hyperactivity co-occurrence is, therefore, important. It might reveal shared causal mechanisms, and it could provide clues for enhanced treatment options, for example, counseling of the comorbid presentation of symptoms instead of the separate treatment of disorders [11].

ADHD and ASD exhibit a male-biased incidence [12]. Stronger androgen exposure can be considered a plausible biological risk factor for both disorders [13]. Indeed, it was reported that high intrauterine testosterone levels may be partially involved in the development of both disorders [14]. For ASD in particular, it was suggested that prenatal testosterone priming may contribute to an “extreme male brain” [15]. Testosterone levels in amniotic fluid were found to predict the level of autistic traits in children at 18–24 months [15], narrow interests at age 4 [16], and gender-typical play at 6–9 years [17]. A peripheral indicator of the exposure to prenatal testosterone in the central nervous system is the 2D:4D finger ratio—the quotient between the lengths of the second and the fourth digits. Accordingly, the greater the exposure and sensitivity to prenatal testosterone and corresponding reductions in estrogens, the greater the likelihood of developing a lower 2D:4D ratio [18]. 2D:4D was found to be smaller in ASD patients, indicating increased prenatal priming in several studies [19, 20]. To the best of our knowledge there are no studies directly examining prenatal testosterone (e.g. in amniotic fluid) in relation to hyperactivity symptoms (or ADHD). However, a study [21] showed that a smaller 2D:4D ratio was more often present in children diagnosed with ADHD compared with those diagnosed with anxiety disorders. Some studies pointed out that actual levels of testosterone also might be elevated in individuals with ASD [22, 23]. Positive correlations have been noted also between salivary testosterone levels and behavioral measures associated with hyperactivity/ADHD such as aggression in disruptive children [24]. Moreover, our initial pilot study [25] in a small sample of pre-pubertal children with ASD revealed positive correlation between hyperactivity symptoms and plasmatic testosterone levels. Nonetheless, results are not always consistent with these theories in individuals with ASD (e.g. [26–28]) or ADHD (e.g. [29–31]).

The androgen activity of testosterone is not only influenced by actual testosterone levels but also by the sensitivity of an androgen receptor determined by the number of CAG repeats in the first exon of the gene encoding the androgen receptor (AR). The number of triplets normally ranges from 11–35 repeats, with a mean of 22 [32].The lengths of this polymorphic chain is inversely related to the transcriptional activity of androgen-dependent genes [33,34]. Thus, the lower number of CAG repeats is responsible for higher transactivational acivity of AR enhancing the androgenic effect. It also has been reported that the amplitude of 2D:4D co-varies with a polymorphic CAG sequence in the gene for AR [35, 36]. Several studies in humans have found that the presence of a smaller number of CAG repeats in the AR gene-(higher sensitivity of AR) was related to ASD [19, 23], ADHD, conduct disorder,oppositional defiant disorder [37] and to violent criminal behavior [38].

Although, hyperactivity symptoms are very often present in individuals with ASD and impose clinical significance, to our best knowledge none of the studies have investigated complex androgen activity (including AR sensitivity and testosterone levels) in relationship to comorbid hyperactivity symptoms in children with ASD.

There is a strong predominance for male gender in children with ASD and increased androgen activity might be a potential factor in the etiopathogenesis of ASD. In neurotypical populations, hyperactivity symptoms (and ADHD) are also very often related to increased androgen activity and show strong male predominance. Therefore, the purpose of this study was to determine the relationship between androgen activity (plasmatic levels of testosterone, sensitivity of AR) and hyperactivity symptoms in boys with ASD. To our best knowledge, this is the first study investigating the relationship between parameters of androgenicity and hyperactivity symptoms in ASD. Given well documented differences in parent and teacher ratings of symptom severity and evidence supporting source-specific syndromes in non ASD [39,40] and ASD [41,42] samples, we employed a standardized parent`s questionnaire (the Nisonger Child Behavior Rating Form) as well as direct examiner`s rating (the Autism diagnostic observation schedule) to assess hyperactivity symptoms in the boys. Since increased androgen activity was found in children with ASD and increased androgen activity is also related to hyperactivity symptoms in neurotypical population, our hypothesis was that increased androgen activity in children with ASD might be one of the biological underpinnings underlying hyperactivity symptoms in this population.

## Methods

### Design of the study and selection of subjects

The study was approved by the Ethical Committee of the Faculty of Medicine, Comenius University (FM CU), Bratislava, Slovakia and has been conducted according to the principles expressed in the Declaration of Helsinki. Inclusion criteria for all subjects in our study were: diagnosis of ASD, ages 3–15 and male gender. Exclusion criteria were any reported endocrinological diseases. ASD children in our study were recruited following diagnostic procedures (in the Academic Research Center for Autism, Institute of Physiology, FM CU) after confirming the diagnosis of ASD (see description of diagnostic procedures below). After one parent signs the consent form, 60 boys with ASD between 3 and 15 years of age were enrolled in our study. Parents then completed the Nisonger Child Behavior Rating Form (NCBRF) and venous blood was drawn from boys with ASD according to standardized procedures (see biological measures below).

The AR gene is located on the X chromosome. Thus males have just one allele giving number of CAG repeats, while females have two [43]. In order to avoid gender bias and have a homogenous group of individuals, only boys were enrolled in this study.

### Behavioral measures

#### Diagnosis of ASD

The diagnosis of ASD was determined in all 60 boys by a clinical psychologist or a psychiatrist according to ICD-10 and DSM-5. The children also underwent behavioral testing by trained examiners at the Academic Research Center for Autism, Institute of Physiology, FM CU. The diagnostic tools involved: observation of a child by the Autism Diagnostic Observation Schedule- second revision (ADOS-2) [44] and the Autism Diagnostic Interview-Revised (ADI-R) [45], a comprehensive interview administered to parents that provides a thorough assessment of individuals with ASD. All children enrolled in the study had to meet the criteria for ASD on both autism scales. The ADOS-2 is a diagnostic tool and a semistructured assessment of social interaction, communication, play, and imaginative use of materials for individuals who may have ASD. It is organized into five modules based on the child’s spoken language level [44]. All the participants were assessed with module 1, 2 or 3. ADOS-2 was administered by trained examiners who were internationally certified to assess ADOS-2 in the clinical and research field and achieved required inter-rater reliability (above 80%). Research indicates substantial inter-rater and test–retest reliability for individual items, excellent inter-rater reliability within domains and excellent internal consistency [internal consistency (Cronbach’s α values) for Modules 1 through 3 were high for the social affect domain (SA) (0.87–0.92) and moderate for the repetitive restricted behavior domain (RRB) (0.51–0.66); test–retest reliability for Modules 1 through 3: SA, RRB, and overall total scores had correlations ranging from 0.68 to 0.92; Inter-rater reliability for SA, RRB, and overall total ranged from 0.79 to 0.98 across the five modules] [44].

#### Measurement of hyperactivity symptoms

Parents of boys with ASD completed the Nisonger child behavior rating form (NCBRF) for intellectual disability [46]. Hyperactivity symptoms were assessed on the hyperactive subscale of NCBRF. The NCBRF for children with intellectual disabilities is a behavior rating scale with good psychometric properties designed for assessment of various behavioral/emotional problems in children and adolescents with intellectual disabilities and ASD [46, 47]. The NCBRF has two Pro-social subscale and six Problem Behavior subscales (Conduct Problem, Insecure/Anxious, Hyperactive, Self-Injury/Stereotypic, Self-Isolated/Ritualistic, Overly Sensitive). The median alpha value for internal consistency was 0.85 for Problem Behavior subscales and 0.78 for the Pro-social subscales [46]. The hyperactive subscale score is the sum of 9 items describing hyperactivity symptoms (e.g., difficulty concentrating, easily distracted, fidgets/wiggles, overactive). Items are rated on a four-point Likert scale. Raters are instructed to consider both the rate of occurrence and the degree to which the behavior was a problem over the last month. Ratings can vary from “did not occur” or “was not a problem” (0) to “occurred a lot” or “was a serious problem” (3).

Hyperactivity symptoms were also assessed during direct observation by trained examiners administrating ADOS-2. Although the ADOS-2 is a diagnostic instrument for ASD, except for items relevant to ASD diagnosis there are also items for assessment of various behavioral/emotional problems observed during a 30–60 mins assessment (e.g. anxiety, overactivity, tantrums/aggression). For our analyses we used an overactivity item that is rated on a four-point Likert scale ranging from absence of problem behavior (0), mild problem behavior (1), moderate problem behavior (2) to marked problem behavior (3). Scores for the item are the same through all the modules.

### Biological measures

#### Plasmatic testosterone

Venous blood samples were drawn from all 60 children into sterile polypropylene tubes containing K2 EDTA (Sarstedt, Nümbrecht, Germany) using standardized procedures the same month of the year from 8:00 to 10:00 a.m. in respect of the circadian [48,49] and infradian [50,51] rhythm of testosterone fluctuations from all children at the Pediatric Department of Children Faculty Hospital CU in Bratislava. Whole blood samples were centrifuged for 5 min at 2000 g immediately after collection. Plasma aliquots were stored at −20°C for not longer than one month. On the day of testing, frozen samples were brought to room temperature and pipetted on to a testing plate. The ELISA assay using a commercial Testosterone ELISA kit was used according to manufacturer's instructions (DRG Instruments GmbH, Marburg, Germany). The intra-assay coefficient of variation was lower than 5% and the inter-assay coefficient of variation was 10%in every measurement. Unfortunately, plasmatic testosterone levels were measured only in 40 boys due to lack of blood sample volumes.

#### Number of CAG repeats- measurement of AR sensitivity

Genomic DNA from whole blood was extracted using the silica membrane based kit (Qiagen, Hilden, Germany) following the manufacturer’s instructions (QIAamp DNA Blood Mini Kit Handbook 04/2010) according to DNA purification protocol for blood/body fluids. The (CAG)n repeat polymorphism in exon 1 of the gene encoding AR was amplified using PCR in 20 μl reaction volume with 250 nmol/L primers: forward:5´ GCGCGAAGTGATCCAGAAC 3´ tagged with 6–carboxyfluoresce in and reverse 5´ CTCATCCAGGACCAGGTAGC 3´, 1× Taq buffer (Fermentas, Vilnius, Lithuania) and 1U of Taq DNA polymerase (Fermentas, Vilnius, Lithuania). The following PCR program was used: initial denaturation step at 94°C for 4 min, followed by 35 cycles each consisting of denaturation at 94°C for 45 s, annealing at 59.5°C for 45 s and polymerization at 72°C for 45 s. The length of the final fragment was 181 bps. The number of repeats was analyzed by capillary electrophoresis. The number of CAG triplets was determined in all 60 boys.

### Data analyses

In order to determine a relationship between biological aspects and behavioral problems statistical analyses were done using IBM SPSS 20 (IBM SPSS 20, Chicago, USA). Before all statistical analyses testosterone levels were log10 transformed in order to achieve a normal distribution.

#### Parent´s questionnaires analyses

Simple linear correlations and simple linear regression, between biological measures (testosterone, number of CAG repeats), behavioral scores on the hyperactivity subscale of NCBRF, and age were conducted.

#### Trained examiner´s rating analyses

Simple linear correlations and simple linear regression, between biological measures (testosterone, number of CAG repeats), behavioral scores on overactivity item in ADOS-2 and age were conducted. To confirm significance after adjustment for age multiple linear regression was used.

## Results

In our study we analyzed biological parameters connected with testosterone and its metabolism in 60 boys of mean age 7.2 ± 3.47 (mean ± SD). Descriptive statistics (mean, median, range, skewness, kurtosis) of measured variables are described in Table 1.

**Table 1:** Descriptive statistics of observed parameters in the whole sample of boys with ASD.

	Mean (SD)	Median	Range	Skewness (Standard error)	Kurtosis (Standard error)
age	7.2 (3.47)	6.5	3–15	0.716 (0.309)	-0.444 (0.608)
hyperactive	13.2 (0.75)	13.0	1–26	0.233 (0.309)	-0.346 (0.608)
overactivity	1.65 (0.14)	2.0	0–3	-0.154 (0.309)	-1.259 (0.608)
TST	0.91 (0.07)	0.75	0.46–2.69	2.024 (0.374)	5.199 (0.733)
CAG	21.35 (3.22)	21.0	11–28	-0.471 (0.309)	1.474 (0.608)

Data on age, hyperactive subscale, overactivity and number of CAG repeats were provided for all 60 (n = 60) boys with ASD.

Plasmatic levels of testosterone were measured in 40 boys (n = 40) with ASD.

*Note*: hyperactive = the Hyperactive subscale on NCBRF

overactivity = Overactive item on ADOS-2 scale

TST = plasmatic testosterone levels in nmol/l

CAG (n) = number of CAG repeats in gene encoding AR

SD = standard deviation

### Parent´s questionnaires analyses

**Fig 1:**
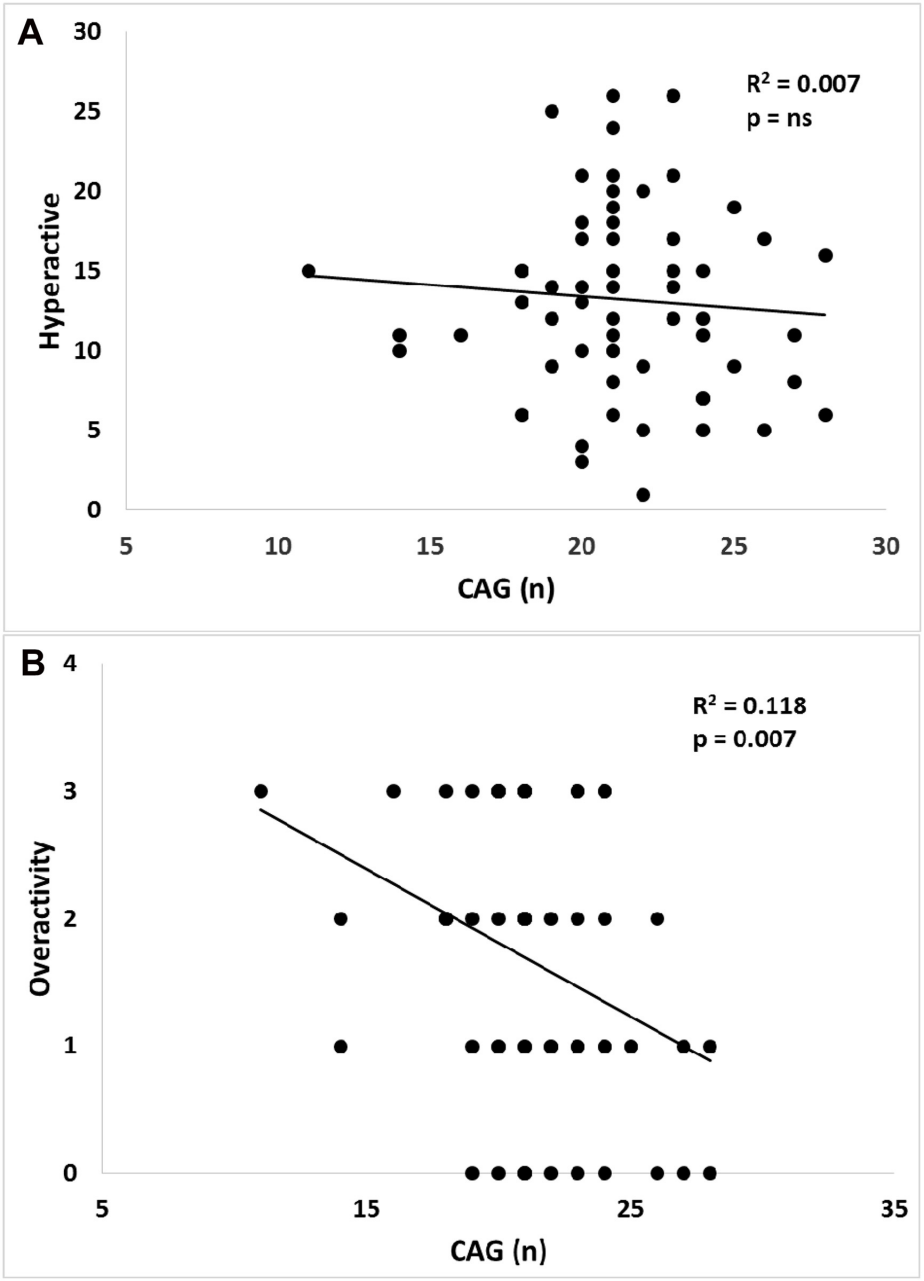
Correlations between CAG (n) in gene encoding AR and hyperactivity symptoms in the sample. (A) CAG (n) was not associated with total score on the hyperactivity subscale of NCBRF (R^2^ = 0.007, p = ns) (B) CAG (n) was significantly negatively correlated with the overactivity score on ADOS-2 (R^2^ = 0.118, p = 0.007).

Correlations between biological measures and behavioral scores on the hyperactivity subscale of NCBRF were assessed. Neither actual levels of testosterone (R = 0.257, R^2^ = 0.066, p = 0.109) nor number of CAG repeats (R = - 0.081, R^2^ = 0.007, p = 0.539) (see Fig 1A) correlated significantly with hyperactivity scores. Age was also not significantly associated with hyperactivity (R = - 0.027, R^2^ = 0.001, p = 0.838) (correlation matrix see Table 2).

**Table 2:** Correlation matrix table including Pearson correlation coefficient (R) and sample size *n* with the variables analyzed in groups of boys with ASD.

		age	hyperactive	overactivity	TST	CAG(n)
age	R	1	-.027	-.389**	.151	-.048
	p		.838	.002	.353	.716
	n		60	60	40	60
hyperactive	R		1	.143	.257	-.081
	p			.276	.109	.539
	n			60	40	60
overactivity	R			1	-.049	-.343**
	p				.762	.007
	n				40	60
TST	R				1	.012
	p					.943
	n					40
CAG(n)	R					1
	p					
	n					

P-values less than 0.05 were considered significant,

** represents p<0.01. Bold font was used for correlations of modest effect size (R>0.3)

*Note*: Hyperactive = hyperactive subscale of NCBRF questionnaire

Overactivity = item score on ADOS-2

TST = plasmatic testosterone level in nmol/l

CAG (n) = number of CAG repeats in gene encoding AR

### Trained examiner´s rating analyses

There was found significant negative correlation between the overactivity score and CAG (n) (R = -0.343, R^2^ = 0.118, p = 0.007) (see Fig 1B) as well as age and the overactivity (R = -0.389, R^2^ = 0.151, p = 0.002).

Association between the overactivity and the number of CAG repeats remained significant also in multiple linear regression with age (overactivity: R^2^ = 0.282, p = 0.002; age: R^2^ = 0.282, p = 0.001).

With a power of 0.8 and significance level of 0.05, our sample size established a cut off value for detectable correlation of R = 0.35, for a power of 0.9 it is R = 0.4.

With a power of 0.8 and significance level of 0.05 the sample size needed to detect strong correlations (R = 0.5) is n = 30, for modest correlations (R = 0.3) n = 85 and for weak correlations (R = 0.1) n = 783.

## Discussion

Although, hyperactivity symptoms are very often present in individuals with ASD, to our best knowledge our study is the first study investigating the role of increased androgen activity (androgen receptor sensitivity as well as testosterone levels) in the etiopathogenesis of ADHD- related symptoms in children with ASD. Our results pointing to associations between parameters of androgenicity (AR sensitivity and plasmatic testosterone levels) and hyperactivity symptoms.

### Androgen receptor sensitivity and hyperactivity symptoms

There was no significant correlation found between parents reported behavioral scores and biological measures of androgen activity. The analysis of trained examiner´s rating revealed that although there was no correlation between plasmatic testosterone levels and overactivity the number of CAG repeats are significantly negatively correlated with overactivity assessed by examiners.

In the present study hyperactivity symptoms assessed by trained examiners were not associated with actual testosterone levels but were strongly associated with a smaller number of CAG repeats which implicates increased AR sensitivity. There was a correlation of modest effect size (R>0.3) and this relationship remained significant even after adjustment for age in multiple regression. Increased sensitivity of AR has been related to increased testosterone effect (androgen activity) and associated with androgen-dependent conditions, e.g. prostate cancer [52] or benign prostate hyperplasia [33]. Morever increased sensitivity of AR was also related to ASD, [19, 23], ADHD, conduct disorder and oppositional defiant disorder [37] as well as violent criminal behavior [38]. Given the focus of our study, although increased plasmatic testosterone levels might not play a role in the etiopathogenesis of hyperactivity symptoms in boys with ASD, the effect of testosterone might be modulated via increased sensitivity of AR that leads to overal increased androgen activity. However there are also studies describing results that are contrary to our hypothesis, showing no significant association between hyperactivity and increased AR sensitivity/androgen activity [29–31].

Moreover, a smaller number of CAG repeats in the AR gene is in some studies associated with increased prenatal androgen exposure as measured by 2D:4D [35,36,53]. This might be indicative of a prenatal organizational effect of increased testosterone on the brain resulting in hyperactivity symptoms. We can speculate that hyperactivity symptoms in boys with ASD in our sample are modulated by increased prenatal testosterone effects (organizational effects) (for review see [21]) resulting in higher AR sensitivity. In the line with this hypothesis, Romero-Martinez et al. [54] found out that masculinized 2D:4D in the parents of ASD children explains some symptoms of ADHD in the parents and in their offspring (for review of the relation between 2D:4D and ADHD symptoms see also [13]). Although some studies describe an association between 2D:4D and the number of CAG repeats, [35,53], reexamination in the larger sample did not prove this genetic association [28]. Similarly, adult circulating testosterone levels did not predict the digit ratio of the left and the right hand respectively [53]. These relationships are rather difficult to replicate and are considered inconclusive. Similarly, we have found no association among three measures of androgenicity (2D:4D ratio, number of CAG repeats in AR and salivary testosterone) [55] not allowing us to shed more light on this controversy. However, measuring 2D:4D as an indicator of prenatal exposure to testosterone, might be additionally conducted in future research in order to assess more specifically a complex (prenatal and actual) androgen effect. Our findings warrant further research in this field in order to bring more clarification to the relationship between the prenatal and postnatal effects of testosterone on hyperactivity symptoms in ASD.

With a power of 0.8 and significance level of 0.05 the sample size needed to detect modest correlations (R = 0.3) is n = 85. Although, one of the major limitations of this study is a small sample size (our sample size is n = 60), our sample is precisely defined. All boys underwent complex psychological assessment based on “gold standard” instruments for diagnosing ASD, administered by trained members of the research team and all the participants met criteria for ASD on both scales (ADOS-2, ADI-R). Moreover, although we employed maximum effort in order to recruit as many boys with ASD in our study as possible, it was not easy to obtain parents informed consent for genetic analysis for more participants.

NCBRF is a behavior rating scale with good psychometric properties and has been widely used in clinical and research practice in populations of children and adolescents with ASD [47]. However in the present study, association between the number of CAG repeats and behavioral scores was found to be significant only for trained examiner`s ratings but not for parent`s ratings of the hyperactivity symptoms assessed by the NCBRF.This discrepancy might be due to several factors. Studies suggest that there is often poor agreement between the parent`s and other examiner`s (e.g. teachers) assessments in general populations as well as in ASD [39–41].This has led Stanger and Lewis [56] to suggest that these ratings are not substitutable for one another.One factor that may explain this discrepancy is that these assessments are often conducted with reference to behaviors observed in different contexts (i.e.the home and the clinical setting, school) (e.g., [57,58], and for those with ASD, in particular [59,60]). Moreover it has been noted that characteristics of the parents may also be associated with discrepancies in the ratings given of problem behaviors. Youngstrom and colleagues [61], noted that parent depression and stress were correlated with disagreements in the ratings of internalizing and externalizing behavior problems compared to teachers.

On the other hand, trained examiners evaluated hyperactivity symptoms during direct observation of the boys within 30–60 mins of ADOS-2 assessment. The goal of the ADOS-2 is to provide “presses” that elicit spontaneous behaviors in standardized contexts [44]. Although, in the assessment the primary focus is on the domains relevant to ASD diagnosis, examiners also are supposed to rate behavioral/emotional problems they observe during assessment. During ADOS-2 assessment examiners rate only actual behavior and do not take into consideration behavioral history (comparing to NCBRF parents assessment). However this assessment is designed to identify a variety of individual behaviors in different standardized contexts (e.g. task, play, conversation) [44] and might be adequate in the clinical judgment of problem behavior such as hyperactivity. Comparing to parent`s ratings, for research purposes, examiners are expected to attend standardized training workshops conducted by workshop leaders and to obtain reliability (above 80%) with workshop leaders as well as within their own research site. Given our results, despite the aforementioned limitations we believe that trained examiner`s ratings might be a more relevant method in searching for an association between biological parameters and hyperactivity symptoms in children with ASD in our study.

Moreover, NCBRF is a broad spectrum assessment instrument used for general assessment of the full spectrum of behavioral/emotional problems. In the next study employing more specific instruments covering the total spectrum of symptoms related to ADHD might be helpful (e.g. SNAP- IV [62]). Moreover, in future research, a behavioral assessment of particular problem behavior in real time [63] might be a useful addition to a detailed parents questionnaire (e.g. SNAP-IV) in order to assess impulsivity and other ADHD-related symptoms [63, 64].

### Testosterone levels and hyperactivity symptoms

In our study, after investigation of the effect of AR sensititivity on hyperactivity symptoms, a parameter sometimes associated with increased prenatal testosterone exposure, we also looked at association between actual testosterone levels and hyperactivity symptoms. Although we did not find an association between plasmatic testosterone levels and hyperactivity symptoms in our sample, some authors suggest that such relationship might exist in a specific age group [65, 66]. Research shows that plasmatic testosterone has a potential role in etiopathogenesis of behavioral problems in puberty [67, 68]. To our best knowledge there are only few studies investigating the relationship between testosterone and problem behavior (particularly aggression and hyperactivity) in children with ASD. In a small study Tordjman et al [66] found out that increased testosterone levels were associated with aggression and this relationship was more obvious for pubertal children, although this association was not confirmed in a larger study conducted by the same researcher [69]. We have also recently found significant correlation between parents ratings of problem behavior (hyperactivity and conduct problems) and testosterone levels in a group of pre-pubertal children with ASD [25]. Moreover, in individuals with ASD some findings support the theory of a precocious puberty expressed by early development of sex organs and increased levels of sex hormones. Majewska et al. [65] found that androgen levels were higher in prepubertal children with ASD compared to the neurotypical control and these anomalies were more prominent in older autistic children (7–9 years old) indicating early puberty. Although in our current sample no association between testosterone and behavioral measures was found in the older prepubertal age group 7–9 (n = 12, R = 0.439, p = 0.153 for parent`s rating, R = 0.139, p = 0.667 for examiner`s rating) or in the pubertal group 10–15 (n = 5, R = 0.451, p = 0.446 for parent`s rating, R = -0.088, p = 0.888 for examiner`s ratings); this might be due to small sample size in these specific age group. Although our study is the first larger study investigating testosterone and AR sensitivity in relation to hyperactivity symptoms the sample size is small, limiting investigation of hormonal effects in a specific age. Another limitation of the study is that testosterone levels were measured only in 40 boys due to lack of blood samples. A major limitation of the current study is also absence of neurotypical control group. As we mentioned before increased androgen activity was described as a potential factor in etiopathogenesis of ADHD itself. Therefore, in order to clarify whether ASD associated with hyperactivity has similar/different biological underpinnings as hyperactivity/ADHD itself, a control group of children with ADHD without ASD also might be helpful in future. Further research with a large sample size in the specific age groups (early puberty and pubertal) and adequate control groups (neurotypical, ADHD) might bring more clarification to the potential influence of actual testosterone levels on behavioral problems in ASD.

Moreover, in our study, we did not find any significant correlation between age and parent`s ratings of hyperactivity symptoms in boys with ASD. This is in contrast to studies conducted in neurotypical populations and in populations of children with intellectual disabilities; they demonstrated strong negative correlation between hyperactivity and age (e.g. [70–73]). However, in line with our results, Lecavalier [10] similarly did not find an association between age and hyperactivity in individuals with ASD using the same scale- NCBRF.

### Conclusions

Despite the limitations of the findings, the results have important implications. Hyperactivity symptoms often reaching a threshold for psychiatric co-morbidity, are very frequent in children/adults with ASD and might have a serious impact on individuals with ASD as well as their families and society. Recently released DSM 5´s criteria for ASD have been considered to be a useful framework to increase the homogeneity of research samples and encourage researchers to identify possible subtypes within ASD. A potential subtype characterized by increased rates of hyperactivity symptoms might have distinct etiopathogenesis and require specific behavioral and pharmacological approach. Considerable evidence exist that increased androgen activity (increased testosterone levels and/or increased AR sensitivity) plays a role in the etiopathogenesis of ASD and ADHD. However, there is insufficient information about the association of increased androgen activity with hyperactivity symptoms in children with ASD. Our study is the first investigating the potential relationship between testosterone, AR sensitivity and hyperactivity symptoms in ASD and proposing an increase of AR sensitivity as a biomarker for a specific ASD subtype accompanied with hyperactivity symptoms. Direct trained examiner`s assessment appears to be a relevant method for evaluation of hyperactivity symptoms in investigating the biological underpinnings of these problems in our study. We also suggest that further research might focus on investigating more complex prenatal (e.g. 2D:4D) and postnatal androgen effects (e.g. activity of enzymes involved in testosterone metabolism) on complex ADHD symptoms in children with ASD. The present study focused on biological factors related to co-morbid hyperactivity symptoms in children with ASD, but further studies should also consider other educative and social aspects such as parenting styles. Future research should also include additional variables such as neuropsychological tests related to multiple domains of executive functions. Our data are relevant and novel as no studies have analyzed AR sensitivity and testosterone levels in relation to hyperactivity symptoms in boys with ASD. Thus, we think that the results should be published and be accessible to other research teams for further studies including complex behavioral and neuropsychological tests. Further analyses are required to determine the involvement and relationship with other important parameters for ASD and ADHD.
